# Supplementary material for: PriME-PGx: La Princesa University Hospital Multidisciplinary Initiative for the Implementation of Pharmacogenetics
Source: J Clin Med. 2021 Aug 24;10(17):3772. doi: 10.3390/jcm10173772 (PMC8432257; doi:10.3390/jcm10173772)
Supplement: Supplementary file 1 [file jcm-10-03772-s001.zip › jcm-1279713-SI.pdf]

Supplementary Material

Supplementary Table S1. Actionable pharmacogenetic tests included in the seven pharmacogenetic profiles and the complete pharmacogenetic report offered at Clinical Pharmacology Department, Hospital Universitario de La Princesa.

**Pain Management Profile**
**CYP2D6**
Tramadol
Codeine
SSRIs (fluvoxamine, paroxetine)
TCAs (desipramine, nortriptiline)
**CYP2C19**
PPIs (omeprazole, pantoprazole, lansoprazole, dexlansoprazole)
SSRIs (citalopram, escitalopram, sertraline)
TCAs (clomipramine, doxepine, imipramine, trimipramine, amitriptiline, nortriptiline)
**CYP2C9**
NSAIDs (celecoxib, flurbiprofen, lornoxicam, ibuprofen, meloxicam, piroxicam, tenoxicam)
**SLCO1B1**
Atorvastatin, simvastatin
**HLA-B*15 y A*31**
Carbamazepine
**Oncology profile.**
**CYP2D6**
Tramadol
Codeine
Tropisetron, ondansetron
Tamoxifen
**DPYD**
5-fluorouracil, capecitabine, tegafur
**TPMT and NUDT15**
Azathioprine and mercaptopurine
**CYP3A5**
Tacrolimus
**UGT1A1**
Irinotecan
**Department of Neuropsychiatry**
**CYP2D6**
Antypsychotics (aripiprazole, haloperidol, risperidone)
SSRIs (fluvoxamine, paroxetine)
TCAs (desipramine, nortriptiline)
**CYP2C19**
SSRIs (citalopram, escitalopram, setrtraline)

**Complete pharmacogenetic report**
**CYP2D6**
Tramadol
Codeine
SSRIs (fluvoxamine, paroxetine)
TCAs (desipramine, nortriptiline)
Tropisetron, ondansetron
Tamoxifen
Antypsychotics (aripiprazole, haloperidol, risperidone)
**CYP2C19**
PPIs (omeprazole, pantoprazole, lansoprazole, dexlansoprazole)
SSRIs (citalopram, escitalopram, sertraline)
TCAs (clomipramine, doxepine, imipramine, trimipramine, amitriptiline, nortriptiline)
Voriconazole
Clopidogrel
**CYP2C9**
NSAIDs (celecoxib, flurbiprofen, lornoxicam, ibuprofen, meloxicam, piroxicam, tenoxicam)
Siponimod
**CYP2C9+HLA-B**
Phenytoin
**SLCO1B1**
Atorvastatin, simvastatin
**HLA-B*15 y A*31**
Carbamazepine
**TPMT and NUDT15**
Azathioprine and mercaptopurine
**CYP3A5**
Tacrolimus
**UGT1A1**
Irinotecan
Atazanavir
**DPYD**
Flucytosine
**HLA-B*57:01**
Abacavir
**IFNL3**
Ribavirin, peg-α-2a/2b interferon
**UGT1A1**
**CYP2B6**

TCAs (clomipramine, doxepine, imipramine, trimipramine, amitriptiline, nortriptiline)	Efavirenz
**CYP2C9**	**CYP2C19**
Siponimod	Voriconazole
**CYP2C9+HLA-B**	**CYP2C9, CYP4F2 and VKORC1**
Phenytoin	Warfarin, acenocumarol
**HLA-B*15 y A*31**	
Carbamazepine	
**Immunosuppressants profile.**	
**CYP3A5**	
Tacrolimus	
**TPMT and NUDT15**	
Azathioprine and mercaptopurine	
**Infectious Diseases profile.**	
**DPYD**	
Flucytosine	
**HLA-B*57:01**	
Abacavir	
**IFNL3**	
Ribavirin, peg-α-2a/2b interferon	
**UGT1A1**	
Atazanavir	
**CYP2B6**	
Efavirenz	
**CYP2C19**	
Voriconazole	
**Gastroenterology profile.**	
**CYP2C19**	
PPIs (omeprazole, pantoprazole, lansoprazole, dexlansoprazole)	
Clopidogrel	
**TPMT and NUDT15**	
Azathioprine and mercaptopurine	
**CYP2C9, CYP4F2 and VKORC1**	
Warfarin, acenocumarol	
**Cardiovascular medicine profile.**	
**CYP2C19**	
PPIs (omeprazole, pantoprazole, lansoprazole, dexlansoprazole)	
Clopidogrel	
**SLCO1B1**	
Atorvastatin, simvastatin	
**CYP2C9, CYP4F2 and VKORC1**	
Warfarin, acenocumarol	

Supplementary File 1. Complete report.

COMPLETE REPORT

Example

PATIENT:	*Example*	PATIENT ID	*Example*
REQUESTING PHYSICICAN	*Example*	DEPARTMENT	*Example*
DATE OF REQUEST	*Example*	REPORT DATE:	*Example*

GENES AND VARIANTS

Gene	Allele	Genotype	Phenotype
CYP2B6	*1, *4…	*1/*6	Intermediate metabolizer
CYP2C19	*1, *2, *17…	*2/*17	Intermediate metabolizer
CYP2C9	*1,*2, *3…	*1/*11	Intermediate metabolizer (AS: 1.0)
CYP2D6	*1, *3, *4, …	*1/*2	Normal metabolizer
CYP3A5	*1, *3, *6	*3/*3	Poor metabolizer
SLCO1B1	*1, *2, *3, *5…		Decreased function
CYP4F2	*1, *3	*1/*1	Normal metabolizer
DPYD	*2, *13, rs67376798, HapB3…	*1/*1	Normal metabolizer
NUDT15	*1, *3	*1/*1	Normal metabolizer
TPMT	*1, *2, *3B, *3C, *4	*1/*1	Normal metabolizer
UGT1A1	*6, *80	*80/*80	Poor metabolizer

Gene	Allele	Genotype	Phenotype
COMT	rs13306278, rs4680	WT/MUT	Intermediate metabolizer
COMT	rs4680, rs13306278	WT/MUT	Intermediate metabolizer
HCP5	rs2395029	T/T	Normal metabolizer
HLA	rs1061235	WT/WT	Non-carrier
IL28B	rs12979860, rs8099917	C/C	Favourable response

For more information, please refer to the Annex, which contains clinical recommendations based on Clinical Pharmacogenetics Implementation Consortium[1] and the Dutch Pharmacogenetics Working Group (DPWG)[2] with the most up to date information at this time.

NOTE: should you have any problems with the interpretation of these results, please contact the Clinical Pharmacology Department of Hospital Universitario de la Princesa: +34915202425 or fcl.hlpr@salud.madrid.org.

PriMEPGx
Princesa Multidisciplinary Initiative for the Implementation of Pharmacogenetics

Supplementary File 1. Complete report.

ANNEX

Gene	Phenotype	Drugs involved	Clinical recommendation
CYP2B6	Intermediate metabolizer	EFAVIRENZ	Clinical recommendations based on CPIC and DPWG pharmacogenetic guidelines written in Spanish.
CYP2C19	Intermediate metabolizer	SERTRALINE, ESCITALOPRAM, CITALOPRAM	Clinical recommendations based on CPIC and DPWG pharmacogenetic guidelines written in Spanish.
CYP2C9	Intermediate metabolizer (AS: 1.0)	CITALOPRAM, ESCITALOPRAM, SERTRALINE	Clinical recommendations based on CPIC and DPWG pharmacogenetic guidelines written in Spanish.
CYP2C9	Intermediate metabolizer (AS: 1.0)	OMEPRAZOLE, LANSOPRAZOLE, PANTOPRAZOEL, DEXLANSOPRAZOLE	Clinical recommendations based on CPIC and DPWG pharmacogenetic guidelines written in Spanish.
CYP2C9	Intermediate metabolizer (AS: 1.0)	CELECOXIB, FLURBIPROPHEN, IBUPROPHEN, LORNOXICAM	Clinical recommendations based on CPIC and DPWG pharmacogenetic guidelines written in Spanish.
CYP2C9	Intermediate metabolizer (AS: 1.0)	CLOPIDOGREL	Clinical recommendations based on CPIC and DPWG pharmacogenetic guidelines written in Spanish.
CYP2C9	Intermediate metabolizer (AS: 1.0)	MELOXICAM	Clinical recommendations based on CPIC and DPWG pharmacogenetic guidelines written in Spanish.
CYP2C9	Intermediate metabolizer (AS: 1.0)	PIROXICAM, TENOXICAM	Clinical recommendations based on CPIC and DPWG pharmacogenetic guidelines written in Spanish.
CYP2C9	Intermediate metabolizer (AS: 1.0)	VORICONAZOLE	Clinical recommendations based on CPIC and DPWG pharmacogenetic guidelines written in Spanish.
CYP2D6	Normal metabolizer	FLUVOXAMINE	Clinical recommendations based on CPIC and DPWG pharmacogenetic guidelines written in Spanish.
CYP2D6	Normal metabolizer	CODEINE	Clinical recommendations based on CPIC and DPWG pharmacogenetic guidelines written in Spanish.
CYP2D6	Normal metabolizer	PROPAFENONE	Clinical recommendations based on CPIC and DPWG pharmacogenetic guidelines written in Spanish.
CYP2D6	Normal metabolizer	HALOPERIDOL	Clinical recommendations based on CPIC and DPWG pharmacogenetic guidelines written in Spanish.
CYP2D6	Normal metabolizer	ATOMOXTINE	Clinical recommendations based on CPIC and DPWG pharmacogenetic guidelines written in Spanish.
CYP2D6	Normal metabolizer	TAMOXIFEN	Clinical recommendations based on CPIC and DPWG pharmacogenetic guidelines written in Spanish.
CYP2D6	Normal metabolizer	VENLAFAXINE	Clinical recommendations based on CPIC and DPWG pharmacogenetic guidelines written in Spanish.
CYP2D6	Normal metabolizer	TRICYCLIC ANTIDEPRESSANTS	Clinical recommendations based on CPIC and DPWG pharmacogenetic guidelines written in Spanish.
CYP2D6	Normal metabolizer	PAROXETINE	Clinical recommendations based on CPIC and DPWG pharmacogenetic guidelines written in Spanish.
CYP2D6	Normal metabolizer	TROPISETRON, ONDASETRON	Clinical recommendations based on CPIC and DPWG pharmacogenetic guidelines written in Spanish.
CYP2D6	Normal metabolizer	METOPROLOL	Clinical recommendations based on CPIC and DPWG pharmacogenetic guidelines written in Spanish.
CYP3A5	Poor metabolizer	TACROLIMUS	Clinical recommendations based on CPIC and DPWG pharmacogenetic guidelines written in Spanish.
CYP4F2	Normal metabolizer	WARFARINE	Clinical recommendations based on CPIC and DPWG pharmacogenetic guidelines written in Spanish.
DPYD	Normal metabolizer	FLUOROPYRIMIDINES	Clinical recommendations based on CPIC and DPWG pharmacogenetic guidelines written in Spanish.
DPYD	Normal metabolizer	FLUOROPYRIMIDINES	Clinical recommendations based on CPIC and DPWG pharmacogenetic guidelines written in Spanish.
UGT1A1	Poor metabolizer	ATAZANAVIR	Clinical recommendations based on CPIC and DPWG pharmacogenetic guidelines written in Spanish.
UGT1A1	Poor metabolizer	ATAZANAVIR	Clinical recommendations based on CPIC and DPWG pharmacogenetic guidelines written in Spanish.
UGT1A1	Poor metabolizer	IRINOTECAN**	Clinical recommendations based on CPIC and DPWG pharmacogenetic guidelines written in Spanish.
IL28B	Favorable response	RIBAVIRIN PEG-IFN	Clinical recommendations based on CPIC and DPWG pharmacogenetic guidelines written in Spanish.

PriMEPGx
Princesa Multidisciplinary Initiative for the Implementation of Pharmacogenetics

Supplementary File 1. Complete report.

SLCO1B1	Decreased function	SIMVASTATIN	Clinical recommendations based on CPIC and DPWG pharmacogenetic guidelines written in Spanish.
SLCO1B1	Decreased function	ATORVASTATIN	Clinical recommendations based on CPIC and DPWG pharmacogenetic guidelines written in Spanish.
TPMT - NUDT15	Normal metabolizer - Normal metabolizer	AZATHIOPRINE, 6-MERCAPTOPURINE	Clinical recommendations based on CPIC and DPWG pharmacogenetic guidelines written in Spanish.
CYP2C9 - HLA	Intermediate metabolizer - Non-carrier	PHENYTOIN	Clinical recommendations based on CPIC and DPWG pharmacogenetic guidelines written in Spanish.
CYP2C19 - CYP2D6	Intermediate metabolizer - Normal metabolizer	AMITRIPTILYNE	Clinical recommendations based on CPIC and DPWG pharmacogenetic guidelines written in Spanish.

REFERENCES

1. Relling MV, Klein TE. CPIC: Clinical Pharmacogenetics Implementation Consortium of the Pharmacogenomics Research Network. Clinical Pharmacology & Therapeutics. marzo de 2011;89(3):464-7.

2. Royal Dutch Pharmacists Association - Pharmacogenetics Working Group, CYP2D6-Tramadol, disponible en: https://www.g-standaard.nl/risicoanalyse/B0001590.PDF.

PriME-PGx

Princesa Multidisciplinary Initiative for the Implementation of Pharmacogenetics

Supplementary file 2. Relevant findings report.

RELEVANT FINDINGS REPORT

Example

PATIENT:	*Example*	PATIENT ID	*Example*
REQUESTING PHYSICICAN	*Example*	DEPARTMENT	*Example*
DATE OF REQUEST	*Example*	REPORT DATE:	*Example*

You have been genotyped for a panel of genes and variants that determine response to numerous drugs. The relevant pharmacogenetic findings are described below:

GENES AND VARIANTS

Gene	Allele	Genotype	Phenotype
CYP2B6	*1, *4, *6, *9	*1/*6	Intermediate metabolizer
CYP2C19	*1,*2, *3, *4, *17	*2/*17	Intermediate metabolizer
CYP2C9	*1,*2, *3	*1/*11	Intermediate metabolizer (AS: 1.0)
CYP3A5	*1,*3, *6	*3/*3	Poor metabolizer.
SLCO1B1	rs4149056	*1A/*15	Decreased function
UGT1A1	*80	*80/*80	Poor metabolizer.

This document requires interpretation by qualified healthcare personnel. Please give it to your practitioner. Should they have any problems with the interpretation of these results, please contact the Clinical Pharmacology Department of Hospital Universitario de la Princesa: +34915202425 or fcl.hlpr@salud.madrid.org.

PriME-PGx
Princesa Multidisciplinary Initiative for the Implementation of Pharmacogenetics
